# Motives of contributing personal data for health research: (non-)participation in a Dutch biobank

**DOI:** 10.1186/s12910-020-00504-3

**Published:** 2020-07-25

**Authors:** R. Broekstra, E. L. M. Maeckelberghe, J. L. Aris-Meijer, R. P. Stolk, S. Otten

**Affiliations:** 1 Department of Epidemiology, University Medical Center Groningen, University of Groningen, PO Box 30.001, FA 40, 9700 RB Groningen, The Netherlands; 2 Department of Social Psychology, Faculty of Behavioral and Social Sciences, University of Groningen, Groningen, The Netherlands; 3 University Medical Center Groningen, Institute for Medical Education, University of Groningen, Groningen, The Netherlands

**Keywords:** Biomedical research, Biobanks, Motives, Participation, Non-participation, Risks, Trust

## Abstract

**Background:**

Large-scale, centralized data repositories are playing a critical and unprecedented role in fostering innovative health research, leading to new opportunities as well as dilemmas for the medical sciences. Uncovering the reasons as to why citizens do or do not contribute to such repositories, for example, to population-based biobanks, is therefore crucial. We investigated and compared the views of existing participants and non-participants on contributing to large-scale, centralized health research data repositories with those of ex-participants regarding the decision to end their participation. This comparison could yield new insights into motives of participation and non-participation, in particular the behavioural change of withdrawal.

**Methods:**

We conducted 36 in-depth interviews with ex-participants, participants, and non-participants of a three-generation, population-based biobank in the Netherlands. The interviews focused on the respondents’ decision-making processes relating to their participation in a large-scale, centralized repository for health research data.

**Results:**

The decision of participants and non-participants to contribute to the biobank was motivated by a desire to help others. Whereas participants perceived only benefits relating to their participation and were unconcerned about potential risks, non-participants and ex-participants raised concerns about the threat of large-scale, centralized public data repositories and public institutes, such as social exclusion or commercialization. Our analysis of ex-participants’ perceptions suggests that intrapersonal characteristics, such as levels of trust in society, participation conceived as a social norm, and basic societal values account for differences between participants and non-participants.

**Conclusions:**

Our findings indicate the fluidity of motives centring on helping others in decisions to participate in large-scale, centralized health research data repositories. Efforts to improve participation should focus on enhancing the trustworthiness of such data repositories and developing layered strategies for communication with participants and with the public. Accordingly, personalized approaches for recruiting participants and transmitting information along with appropriate regulatory frameworks are required, which have important implications for current data management and informed consent procedures.

## Background

There is an increasing collection and transmission of personal data in society. Biobanks and other large-scale, centralized data repositories are a special form of data collection, storage, and use of large volumes of diverse personal data obtained from patients and citizens. Their aim is to contribute to the development of research activities in medicine [1–3]. They constitute valuable resources for healthcare professionals and facilitate innovations in epidemiological, genetic, and public health research [2, 4]. These data repositories facilitate the efficient performance of large-scale and continuous data analyses and enable researchers and clinicians to generate individual tailored clinical care [5–9]. Contributing personal data to biomedical research must be voluntary [9–14].

There is widespread consensus among European researchers that contributions of personal data by citizens foster scientific development and are therefore commendable [14]. Studies show that participants also share this consensus. They are motivated to do good deeds by helping researchers, patients, and doctors [2, 15–17]. A lot of citizens nonetheless choose not to contribute personal data for medical scientific research purposes, since percentages of unwilling citizens in representative samples range from 17 to 44% [18, 19]. This raises the question why some individuals do (participants) and others (non-participants) do not actively contribute their personal data to data repositories for medical scientific research, such as population-based biobanks.

The current literature mentions two types of risk that might explain the decision to participate or not in medical scientific research [2, 15]. The first type are the foreseeable risks associated with repositories, such as increasing loss of direct control over personal data, loss of privacy, or data breaches. The second type includes less foreseeable risks, for example the risk of unauthorised re-identification with potentially harmful consequences that is incurred as a result of linking different data repositories for the conduct of new kinds of analyses [2, 17, 20]. This second type of risk is, in contrast to the first, a risk that is rarely addressed in currently used consent forms yet exists [2].

The two types of risk offer however insufficient explanation why some participants, which we refer to as ex-participants, withdraw during the data collection process. Several studies have suggested that withdrawal or other non*-*participation is motivated by perceptions of the consequences of helping others. For example, concerns regarding the commercialization of public research that derogates social benefits intended for others [21–23], risks of privacy violations following individual and group data sharing [2, 19, 22, 24–34], or potential discrimination [35]. More insight is needed in what factors drive this behavioural change. A comparison of the experiences of ex-participants of a biobank and non-participants with the views of participants could therefore yield insights into the reasons for participation, non-participation, and, in particular, withdrawal.

Our aim is to understand how motives, values, and expectations can differ in participation and non-participation. This understanding of underlying mechanisms could yield insights on variance in ethical understandings of biobank research, improvement of research data governance in biobanking, and effective strategies of participant recruitment in biomedical research. In this qualitative study of ex-participants, participants, and non-participants of a Dutch population-based biobank, we investigated and compared the views of participants on contributing data to the biobank with those of non-participants and ex-participants about why they chose not to participate and why they ended their participation respectively.

## Methods

### Data collection

We conducted in-depth interviews with individuals who were ex-participants, non-participants, or participants in Lifelines, a Dutch population-based biobank, to explore the motives underlying their participation and non-participation in a centralized health research data system. Lifelines is a multidisciplinary, prospective population-based cohort study that entails a unique three-generational design for examining the health and health-related behaviours of individuals living in the Northern Netherlands. Lifelines encompasses a broad range of investigative procedures used to assess the biomedical, sociodemographic, behavioural, physical, and psychological factors that contribute to health and disease within the general population, with a special focus on multi-morbidity and complex genetics [36]. The recruitment of participants began in 2006 and continued up to 2013. Recent year, Lifelines launched an initiative introducing innovative big data technologies that include, for example, providing research participants with personalized feedback.

We applied a narrative interview approach with a tailored topic guide that was partly derived from the DIPex methodology that allowed for a discussion, clarification, and verification of unanticipated themes [37, 38]. The topic guide, as shown in Table 1, was developed in a research project about research (non-)participation and big data consisting of the current study and a qualitative study about trust [35]. Six relevant themes relating to the motives, values, and expectations associated with participation and non-participation were identified in this topic guide, based on the state of knowledge evidenced in the scientific literature on (non-)participation in biobanks, public goods, trust, and data sharing: (1) becoming a (non-)participant; (2) objective aspects of participation (e.g., participation overall, tasks, and feedback); (3) subjective aspects of participation (e.g., expectations of accomplishment and feelings of identification); (4) definitions of and attitudes towards big data; (5) perceived benefits and threats associated with big data; and (6) decisions to provide personal data. The topics discussed in the interviews were derived from these themes.

**Table 1:** Interview topics and illustrative questions

Topic	Example questions
Association with biobank	How did you get involved?
Decision of (non-)participation	What were your considerations?
Reality	Was your experience as expected or not?
Future	Will you participate in Lifelines or in another centralised large-scale research project in the future?
Overall perception	How is participation in Lifelines perceived in general?
Tasks	How are the tasks related to participation in Lifelines perceived?
Feedback	What do you think of the feedback on your data you (would) receive?
Outcome hoped for	What motivates individuals to participate?
Benefits	What are the benefits and opportunities relating to participation?
Disadvantages	What are the risks and disadvantages relating to participation?
Definition	What are centralised large-scale data repositories?
Attitudes towards large-scale centralised data repositories	Are the public’s attitudes towards collection, linking and use of this data generally positive or negative?
Benefits of large-scale centralised data repositories	What are the benefits of collecting, linking and using data?
Risks posed by large-scale centralised data repository	What risks are posed by the collection, linking and use of data?
Personal balance of benefits and threats	From your personal perspective, how are these considerations balanced?
Societal balance of benefits and threats	From a societal perspective, how are these considerations balanced?

All of the interviews were conducted by a member of the research team (RB), who is a trained and experienced interviewer. Each interview covered all of the topics included in the guide and lasted between 30 and 65 min. Data saturation was reached, when informational redundancy was achieved in the interviews [39]. Current participants of Lifelines were interviewed following their regular visits to the Lifelines facility. Most of the interviews were held at the University Medical Center Groningen. All four ex-participants and eight out of fifteen non-participants were interviewed at their homes on their request. All respondents provided verbal informed consent for their participation and the recording of the interview. This consent is documented by recording on video or writing. Apart from one interviewee, who only consented to a written recording of the interview, the remaining interviewees consented to audio recordings of their interviews. All of the audio recordings were transcribed by an independent professional organization. These recordings were anonymized and stored at the highly secured servers of the University Medical Center Groningen. The access to these servers is only with permission of the researchers and excludes access for staff of Lifelines. The Medical Ethics Review Board of the University Medical Center Groningen exempted this study from the ethics review according to the Medical Research Involving Human Subjects Act of the Netherlands, as it did not involve collection of any health-related data.

### Recruitment of respondents and sampling

A maximum variation sampling strategy was applied to recruit participants for the study [40]. Thus, we ensured that the sample of selected interviewees was heterogeneous [41, 42]. Between August and September 2016, we interviewed 36 individuals: 17 participants, four ex-participants, and 15 non-participants in the Lifelines biobank. We refer to these distinct groups throughout this paper as ‘interviewees’ while distinguishing ‘participants’, ‘ex-participants’, and ‘non-participants’. Table 2 shows the characteristics of all of the interviewees. Their average age was 45 years (ranging between 20 and 68 years) and 17 out of the 36 interviewees were male.

**Table 2:** Respondents’ Characteristics

Respondent	Sex	Age	Occupational Field	Participation status
1	Female	65–70	Human resources	Participant
2	Male	40–45	Transportation	Non-participant
3	Female	60–65	Education	Non-participant
4	Male	30–35	Legal and social work	Participant
5	Male	50–55	Photography and architecture	Ex-participant
6	Male	55–60	Municipality	Non-participant
7	Female	35–40	Home maintenance	Ex-participant
8	Male	25–30	Warehouse maintenance	Participant
9	Female	55–60	Retail	Participant
10	Male	30–35	Entrepreneur	Participant
11	Male	20–25	Student	Participant
12	Female	50–55	Law	Participant
13	Male	25–30	Student	Non-participant
14	Male	65–70	Mechanical engineering	Participant
15	Male	55–60	Home maintenance	Non-participant
16	Female	25–30	Publisher	Non-participant
17	Female	45–50	Entrepreneurship	Non-participant
18	Female	65–70	Retired	Participant
19	Male	50–55	Management	Participant
20	Female	25–30	Energy policy	Non-participant
21	Female	35–40	Healthcare	Participant
22	Female	50–55	Healthcare	Participant
23	Female	25–30	Human resources	Non-participant
24	Female	45–50	Education	Participant
25	Male	40–45	Municipality	Participant
26	Female	65–70	Farming	Non-participant
27	Female	40–45	Librarian	Ex-participant
28	Female	50–55	Social work	Ex-participant
29	Female	35–40	Management	Participant
30	Male	35–40	Engineering	Non-participant
31	Male	55–60	Human resources	Non-participant
32	Female	30–35	Home maintenance	Non-participant
33	Male	40–45	Management	Participant
34	Female	60–65	Retired	Participant
35	Male	35–40	Business development	Non-participant
36	Male	45–50	Entrepreneurship	Non-participant

We recruited individuals partly from the Lifelines biobank. To ensure their privacy, Lifelines recruited participants and ex-participants of the biobank without the researchers being aware of their identity. Ex-participants who had terminated their participation in the few months prior to the commencement of our study were approached by phone, using a protocol created by the researchers (RB and JA) during the final stage of Lifelines’ established withdrawal procedure. Moreover, the Lifelines biobank also invited non-participating partners of participants who had contributed to the biobank. Ex-participants were the least willing to participate in our study. Although 90 people were invited, only four (4.44%) consented to participate. Reasons given for not participating in our study were ‘no interest’, ‘not available’, and ‘done with participating’. The sample of ex-participants was too small to enable generalization, but yielded the in-depth information that we were seeking. Non-participants were recruited through direct encounters with members of the public at the entrance of Groningen’s central public library and by personal invitation vis-à-vis various social networks as well as through online and offline platforms.

### Data analysis

We analysed every phrase in each of the 36 interview transcripts within the context of the entire interview, and, where appropriate, a code pertaining to its content was generated or assigned. Codes could be applied multiple times within each transcript, and phrases could comprise multiple codes. Codes with related content were clustered within groups that were subsequently thematically categorized. An initial coding protocol containing a description of the codes, groups, and themes was developed based on a close reading of three transcripts. This coding protocol was evaluated through an iterative process, whereby two or three researchers cross-checked analyses of each of the transcripts. Once a consensus regarding the content of the codes, groups, and themes had been reached, the resulting coding protocol was used for the remaining transcripts. Five researchers coded the transcripts. Six random transcripts were coded by at least three researchers. This procedure ensured agreement among the researchers regarding the coding. Subsequently, one researcher coded all of the remaining transcripts.

The results of the interviews were categorized according to four themes. One of these themes, ‘perception of big data’, that emerged from our topic guide was selected because it was a focal theme during the interviews prior to the analytical phase. The other themes did not emerge directly from our topic guide but instead featured prominently in the interview data. This approach can be considered as valid, as the adaptive theory for qualitative research allows for the influence of theory on research [43]. Transcripts were primarily analysed using the computer-assisted qualitative data analysis package, Atlas TI, version 8, to retrace and evaluate quotes along with their codes, groups, and themes [44].

## Results

We distinguished five themes relating to the motives, values, and expectations of ex-participants, participants, and non-participants in our dataset. The first three themes pertained to views and motives regarding the initial or current participation of ex-participants and participants. The last two themes touched on the ex-participants’ views and motives relating to their withdrawal and on non-participants’ views.

### Contribution to the public good: science, healthcare, and society

Initially, most of the participants perceived their participation in Lifelines in terms of the donation of their time, information, and data to a public good, namely health. They believed that they were helping society by voluntarily investing time and effort, which they could also do in other ways. They compared their contribution of personal data to Lifelines to a gift bequeathed to a charity or to organ donation. For example, one participant (P33) explained: ‘My role is not to conduct research but to provide things [such as information, money, or data]’.

Ex-participants emphasized that the central motive for their initial participation in Lifelines was their desire to contribute to society. According to them, the purpose of research was to ‘gain insights’ and to ‘create innovation in society’. Therefore, in their view, participation would facilitate societal progress. An ex-participant (EP28) explained this as follows: ‘There was at that time no reason to say “no”. It was about gaining collective insights into the world; you need people and you need a research group’.

This perception of contributing to societal progress was fostered through the awareness that Lifelines is a prospective cohort and a large-scale, centralized data repository. Ex-participants argued that the scale and length of the Lifelines data collection was unique and could thus provide new insights for medical sciences in the long term. Their initial expectations were that the results and implications of the research conducted using Lifelines data would primarily benefit future generations. These expectations were similar to those of the participants. Two participants explained this as follows:

I think I have little benefit, I don’t know. I think it’s for the generation after me, for which I’m participating in now.. .. My parents participated in such events for us, and so I am participating for the next generation. (P22).

Research must be useful for society as a whole [so] that we ultimately flourish or become less ill. .. so that things get better; so that we leave it better than we found it. It is that idealistic idea so to speak. (P1).

For the participants, the primary motive for participation was to contribute to science. Moreover, all of the participants and ex-participants subscribed to the belief that participation would or could facilitate scientific progress and specifically the progress of the medical sciences. Participants as well as ex-participants explicitly stated that they highly valued scientific research in general. Participants were primarily interested in improving health and medical knowledge because they prioritized health as a profoundly important value. They perceived their donations of personal data as contributions to health-related research that was aimed at facilitating a better understanding of the development and course of illnesses. One participant (P22) expressed the view that ‘the medical sciences can’t stagnate, so you must cooperate in order to help people to do research and make it better.. .. That is what my aim is’.

### Participation to help family members or friends

Similarly, a motive for participation mentioned by the interviewees, especially ex-participants, was ‘helping family or friends’, which was described as an investment of time and effort to benefit the current generation of family members or friends. They perceived this as an important motive influencing their decision to participate. An ex-participant (EP27) made the following statement:

As I recall, I participated because a family member participated too. It was probably my mother, but I am not sure.. .. We spoke about the (Lifelines) research on health and diseases at that time. However, exactly how that went I don’t recall because of the passage of time. I started participating after that conversation.

Several current Lifelines participants who were recruited by their friends or family members considered participation in the programme to be almost self-evident, given the objective of Lifelines to follow the trajectories of three generations. A participant (P18) explained this as follows:

Well, we have two daughters; one daughter took part in it and so did her son. So, I thought, “then someone from our generation will have to participate too”, so that they will have someone of my generation and get three generations in total. My husband didn’t want to participate, so I joined.

### Contributing to obtain individual benefits

The prospect of a ‘medical check-up’ was also a motivating factor for the contributions of ex-participants. Once every 5 years, participants visit a Lifelines location, where they provide blood samples and are subjected to physical measurements (e.g., those relating to anthropometry, electrocardiogram, and spirometry). Afterwards, some of these measurements are returned to the participants. Interviewees perceived this feedback as a free medical check-up that provided them with extra health-related information. These same results also sent to the general practitioner (GP) with a recommendation on whether the GP should actively contact the participant to arrange further clinical investigations.

Although participants highly appreciated this sharing of data, which they valued as a supplementary bonus to their personal healthcare, most of them emphasized that it was not the primary motive for their participation. They valued some of the other individual benefits as well, such as ‘acquiring knowledge in general’, ‘learning about themselves’, and ‘the joy of gaining insights into the scientific process’. Despite the fact that all of the participants were informed that not all of the measured data would be returned and that the collected data would not be screened for diseases, they felt that the chances of obtaining an early diagnosis of illness would increase through their participation in the programme. They also assumed that they were free of any illness if their GPs did not recommend further investigations. One participant (P19) noted:

I do like it. Suppose I am not in good health; it is of course out of self-interest as well, to find out at the earliest possible stage. Look, normally if you don’t have anything, then you don’t go to a doctor. Nevertheless, here an EEG is done, your blood is measured, your urine is measured, and other things are done. So if there is something wrong. .. then hopefully you will know at an early stage. So, that is also one of the reasons for me [to participate].

A few participants thought that their own data could prove useful if they were later to fall ill. Participation would therefore enable them to keep records of their personal health. For example, according to one participant (P11), ‘it is a sort of back up. We [my GP and I] can look into the past, if I feel ill or have some symptoms. Perhaps I [my GP and I] can use it?’ Applying this line of reasoning, some of the participants specifically observed that their participation and procurement of data could provide their offspring with extra health-related information.

### Not contributing to a public good

Prior to their withdrawal from the programme, ex-participants considered the large-scale, centralized data repository to be a public good. However, this positive perception later changed to suspicion, as they felt that their personal data would be sold or misused. The new perception was driven by sceptical news items, negative perceptions of family members and friends, or a negative experience during a research visit. They mentioned that their concerns about the data repository being used by insurance companies or by the government to exclude people collectively and individually were among the primary reasons why they withdrew from the Lifelines programme. Another reason pertained to their belief that Lifelines was a profit-oriented organization and, therefore, their participation did not contribute to a public good.

Non-participants also considered the risk of collective exclusion with Lifelines to be plausible. For example, they noted the effect of personalized information on insurance premiums that could derogate a ‘social society’. Some of the non-participants pointed to historical evidence of the isolation and exclusion of social groups resulting from the misuse of comparable repositories or systems. One non-participant (NP36) specifically referred to the civilian administrative system that prevailed in the Netherlands prior to and during World War II: ‘ … they used to register the religions of civilians. That is not done any more for good reasons. These practices originate from World War II and the [treatment of the] Jews. Those [data] were used to trace them’.

Similar to ex-participants, non-participants generally thought that their participation in large-scale, centralized data repositories could prompt a decrease in life opportunities for themselves and others through decisions on, for example, mortgages or governmental support. One non-participant (NP6) elaborated on this point as follows:

Well look, if it [the data] is linked to healthcare insurance, they could potentially determine from this information that this male or female has a risk that is above average. Therefore, they may ask for a higher premium or deny the individual insurance. That is. .. I perceive it as a definite risk and threat if such organizations are involved.

Differing from ex-participants, however, non-participants ranked the type of research compiled for population-based biobanks lower than other types of research in terms of its importance. Several non-participants were not particularly concerned about health research, showing considerably more interest in topics relating to economics or energy-related innovations. Although they saw some use for research facilitated by Lifelines data, they did not feel that contributing their personal data would be of much use, as a non-participant (NP20) explained:

I don’t necessarily perceive it [as being] negative; nor do I immediately think: “oh yes, I am going to participate”. I think that it depends very much on. .. as you explained it now, it [research participation] doesn’t attract me. I don’t think: “oh yes I have to participate, because I can be of use” or something like that.

### A lack of individual benefits

Ex-participants explained that one of the reasons for their withdrawal was that they did not perceive their participation as being useful in terms of procuring personal benefits that matched their expectations. They discovered that participants could still become ill regardless of their participation in Lifelines, indicating a lack of individual benefit of a ‘medical check-up’. Although non-participants also considered potential individual benefits such as a ‘medical check-up’ or gaining ‘part of the profit’, they did not especially value or see the individual benefits gained from participating in Lifelines. This lack of benefits for themselves did not motivate them to participate in the biobank. One non-participant (NP31) explained this as follows:

It must clearly matter in my view. For a multi-year research project, one can claim that it is of use. Of course, it is of some use. However, it is difficult to grasp how long it will [continue to] be of use.. .. Of course, if so much data is compared, your data will disappear in a large dataset.. .. Then you get research results from which you must filter out the personal implications. In that sense, for the larger group, it is wonderful that many people are participating. Nevertheless, at this point, I am not interested in participating for this reason [lack of individual benefit].

Whereas some participants and ex-participants expressed the view that personal health experiences featured in their decision to participate in Lifelines, some of the non-participants explicitly stated that they had not had any immediate experiences relating to health issues themselves or within their families. Therefore, they did not perceive the active contribution of personal data as being imperative. As one non-participant (NP23) explained, ‘I don’t know any people with chronic diseases.. .. I need some personal benefit. I can imagine that with Lifelines, you get knowledge about your own health and body. So either that or I am helping someone within my circle’.

Moreover, non-participants mentioned that the burden of investing time for each visit, and for the duration of participation, and the effort expended in relation to the complexity of the performed tasks were also motives for their non-participation. Non-participants found it difficult to acquire an overview of these burdens or costs and of the implications of participating in a population-based biobank. Some of them were hesitant about participating because they were apprehensive about making a long-term commitment to such a project.

## Discussion

In this study, we investigated and compared the views of participants and non-participants on contributing to a large-scale, centralized health research data repository with the perceptions of ex-participants who decided to end their participation. We explored the motives, values, and expectations of ex-participants, participants, and non-participants of a Dutch population-based biobank encompassing three generations. Participants and non-participants evidenced different perceptions on how they could help others through the contribution of their personal data for biomedical research. Nevertheless, they shared similar intentions about contributing to society and science and helping family members and friends. Participants and non-participants did not agree on realistic risk levels relating to participation. Thus, non-participants and ex-participants expressed more concern relating to participation in large-scale, centralized research data repositories as well as threats of public institutions to constitutional rights. Several studies conducted in Europe and in the United States have confirmed such differences between participants and non-participants in terms of attitudes towards risks and levels of trust, especially in relation to genetic research [19, 23, 26, 32, 45–47].

Our findings showed that intrapersonal differences might exist between participants and non-participants relating to their considerations on trust in society, and in data repositories, as well as their perceptions about society and research. These findings therefore provide some support for significance of the relationship between trust and willingness to participate in research that is well-established [18, 19, 23, 26, 45–47]. Although a lack of trust is associated with high levels of concern and low levels of awareness of current research and data-sharing or data-linking practices [18, 19, 47, 48], our findings indicate that simply increasing the provision of information on data management or biobanking might indeed not be enough [18].

Specifically, our findings show that a lack of engagement in health research practices and inadequate communication of information do not fully explain non-participation in biobanking, as perceptions about society and research appeared to vary between participants and non-participants. Recent quantitative studies conducted on individuals unwilling to contribute to biobanks confirmed that their willingness to participate depended on their perceptions and interest or familiarity in biobanking [18, 28]. Moreover, the findings of a pan-European quantitative study and of a systematic review revealed that a preference for broad or narrow informed consent depended on attitudes towards biobanking research, such as benefits, concerns, and information needs [19, 48]. These diverse perceptions of benefits and risks are not confined to potential participants. A recent systematic review confirmed that there were marked variations in the perceptions of members of expert groups and organizations regarding pertinent ethical principles and regulatory norms governing the sharing of international health research data [49].

The formulation of objective criteria for the regulation and the use of personal data is therefore essential in biomedical research and for large-scale, centralized data repositories to counter prejudices based on misunderstandings of risks and benefits of biobanking. In other words, the focus of regulation and use of personal data should be on enhancing trustworthiness of data repositories to provide objective information about the biobanking context and addressing concerns of participation and non-participation [50]. This is the reason why criteria relating to organizations’ trustworthiness need to be ingrained within current regulatory and informed consent procedures and implemented more widely, especially in the areas of learning health systems and personalized medicine that entail continuously changing data [50]. For example, a study conducted in Finland revealed that the contexts in which donations were made to a blood bank for patients or to biobanking could trigger different concerns and attitudes [46]. The accounting for context becomes even more key in the case of a large-scale, centralized data repository because of the increasing morally ambiguous and indirect consequences of participation in big data health research for society and for participants [6, 51]. For example, debates about insights in genetic test results and providing feedback to individuals and their families about the genetic risks are ongoing, given differing views on the ‘soft’ impacts of genetic risks considered as a good practice [52, 53]. An effective regulatory framework should clearly stipulate the responsibilities of data repositories in relation to health research while outlining the implications of participation and non-participation. The findings of several European studies confirm that the trustworthiness of the biobank and of the biobanking context critically influence individuals’ decisions to contribute their personal data [19, 32, 46, 48].

A practical implication that follows from a more nuanced understanding of the motives behind participation and non-participation in large-scale, centralized data repositories relates to the need to acknowledge the important influence of intrapersonal factors, such as values, trust in society, and interest. The adoption of a more personalized approach in relation to potential participants could be facilitated through the development of new models for communicating information and recruiting participants for health research [54, 55]. A limited focus on facts and consent within the prevailing generalized information and communication procedures leads to the oversimplification of researcher–participant interactions [8, 52] that does not address the need for more layered and informative interactions in the contemporary context of data collection and research. Our findings revealed that issues of individual or societal benefits and risks importantly influenced the respective decisions of ex-participants and non-participants to withdraw or not to participate at all. The findings of a recent systematic review of similar studies showed that the public welcomes information about current research and data-sharing practices [48]. Therefore, training programmes developed for personnel who recruit individuals to contribute to large-scale, centralized data repositories along with the provision of a comprehensive explanation of current practices and potential future implications of participation to participants and non-participants are important future tasks.

A notable strength of the present study lies in its in-depth investigation of motives for (non-)participation. Moreover, by including participants, non-participants, and ex-participants in our study, we have been able to provide a broad overview of the motives for (non-) participation in large-scale, centralized health research data repositories. However, this study also has some limitations. Firstly, the samples, especially of ex-participants, were relatively small. Consequently, some important motives for ending participation may have been missed despite the samples’ heterogeneity. For example, it is likely our study lacks the voices of ex-participants and non-participants not consenting due to limited time available or a strong aversion of scientific research. Secondly, we only investigated (non-)participation pertaining to a large-scale, centralized data repository focusing on health research. Therefore, our findings and conclusions are bound to the specific context of health research. Their applicability to other settings, such as sustainability research or consumer services, requires further investigation.

## Conclusions

In conclusion, we investigated and compared the motives underlying participation, non-participation, and withdrawal of participation in the context of a large-scale, centralized population-based biobank in the Netherlands. Our findings revealed that motives for (non-) participation are complex and that intrapersonal characteristics may be important influencing factors. Acknowledgement of this complexity and the pursuit of personalized approaches relating to recruitment and information communication procedures, along with effective regulatory frameworks, could strengthen collaborative research and healthcare initiatives.

## Data Availability

The dataset used and/or analysed during the current study is available from the corresponding author on reasonable request.
